# Drug Induced Liver Injury Attributed to a Curcumin Supplement

**DOI:** 10.1155/2019/6029403

**Published:** 2019-10-20

**Authors:** Zaid Imam, Majd Khasawneh, Diana Jomaa, Hira Iftikhar, Ziad Sayedahmad

**Affiliations:** ^1^Departments of Internal Medicine, William Beaumont Hospital, Royal Oak, MI, USA; ^2^Oakland University William Beaumont School of Medicine, Royal Oak, MI, USA

## Abstract

More severe reactions, higher acute liver failure rates, and higher recurrence rates on re-challenge occur with supplement-related Drug Induced Liver Injury (DILI) (Medina-Caliz et al., 2018). We report a case of curcumin-induced hepatocellular DILI in a 78-year old female admitted with jaundice, with a one-month latency. Extensive evaluation for alternative etiologies of hepatotoxicity was unremarkable. The Roussel Uclaf Causality Assessment Method (RUCAM) score of 6 for the supplement indicated a probable association (score >8: highly probable association). Peak levels of aspartate aminotransferase (AST) and alanine aminotransferase (ALT) were >20 times upper limit of normal. A 48% decrease in AST and ALT levels was observed 7 days after discontinuation of the supplement, and resolution of transaminitis was observed in 42 days. No re-challenge was performed. In conclusion, this case emphasizes the importance of recognizing curcumin supplements as DILI triggers. Furthermore, it reiterates the need for careful evaluation of herbal and dietary supplements (HDS) consumed by patients to identify potential DILI culprits, and to ultimately prevent DILI reactions with significant morbidity and mortality.

## 1. Introduction

It is challenging to diagnose DILI by primary care providers and specialists, as numerous alternative etiologies must be first excluded. Population surveys in France and Iceland estimate annual incidence between 14 and 19 per 100,000 inhabitants of DILI [1, 2]. Herbal and dietary supplements (HDS) accounted for 16% of DILI reactions in the Iceland population cohort [2]. In a Spanish DILI registry, HDS-associated DILI reactions had higher rates of acute liver failure than DILI caused by prescription medications and recurrence rates on re-challenge were higher [3].

Curcumin supplements are one of the most consumed herbal supplements in the United States (U.S.) [4] and have shown some promise in the treatment of osteoarthritis [5]. Curcumin is extracted from the plant turmeric (*Curcuma longa*). The content of curcuminoids in turmeric powder is 3.14%, which is used as a spice in cuisines of the Middle East and Indian subcontinent [6]. Much higher amounts of curcumin are present in over the counter supplements. This manuscript presents a case of Grade 3 DILI (severe hepatotoxicity) [7] attributed to a curcumin supplement with complete recovery on discontinuation of the supplement.

## 2. Case Presentation

A 78-year old Caucasian female with a history of well-controlled type 2 diabetes mellitus, and essential hypertension reported the presence of jaundice and acholic stools for one week. She denied any fever, abdominal pain, pruritus or changes in her bowel habits. She denied any consumption of alcohol as well as the use of any analgesics or antibiotics. She also denied any history of travel outside Michigan, United States of America. Her medications included aspirin (81 mg), citalopram, losartan, metformin, and oxybutynin, all of which she has taken for at least one year. She also took simvastatin for 2 years with no adverse effects but had replaced it a month prior to her presentation with a once daily over the counter curcumin supplement without seeking medical advice.

On physical examination, her blood pressure was 126/65 mmHg, her heart rate 66 beats/min, and her temperature 36.6°C. She appeared in no acute distress and was jaundiced. No hepatosplenomegaly, ascites, asterixis, encephalopathy, or other stigmata of chronic liver disease were noted on physical examination.

Her laboratory results showed a white blood cell count of 5,300 cells/mm^3^ with a normal differential, hemoglobin of 12.6 g/dl, platelet count of 282,000 cells/mm^3^, blood urea nitrogen of 11 mg/dl, creatinine of 0.59 mg/dl, alkaline phosphatase (ALP) of 171 U/L (lab normal: 33–120 U/L), aspartate aminotransferase (AST) of 581 U/L (lab normal: 0–34 U/L), alanine aminotransferase (ALT) of 609 U/L (lab normal: 9–47 U/L), total bilirubin of 12.8 mg/dl, direct bilirubin of 7.4 mg/dl, international normalized ratio (INR) of 1.1, serum albumin of 4.5 g/dl, and thyroid stimulating hormone (TSH) level of 1.99 mU/L. Liver chemistries from 5-years ago were normal (ALP: 101 U/L, AST 13 U/L, ALT 19 U/L, total bilirubin 0.3 mg/dl). Her R-factor was calculated at 15 consistent with a hepatocellular pattern of liver injury. Hepatitis A, B, C, and cytomegalovirus serologies were negative. Epstein-Barr virus and herpes simplex virus-1 serologies indicated previous exposures. Hepatitis E testing was not obtained given the patient had no exposure to jaundiced individuals, no recent travel history, and lived in a nonendemic country. Serum IgG level was 951 mg/dl (lab normal: 550–1650 mg/dl) with normal subclass levels. Antinuclear antibody (ANA) titers were 1 : 320 with a speckled pattern, and anti-smooth muscle antibody (ASMA) titers were 1 : 20. Anti-LKM (liver-kidney-microsomal) and antimitochondrial antibodies (AMA) were negative. Ferritin level was 689 ng/ml with a transferrin saturation of 32%. An abdominal ultrasound demonstrated a normal hepatic echotexture and incidental cholelithiasis. A magnetic resonance cholangiopancreaticogram (MRCP) revealed no additional findings.

**Figure 1:**
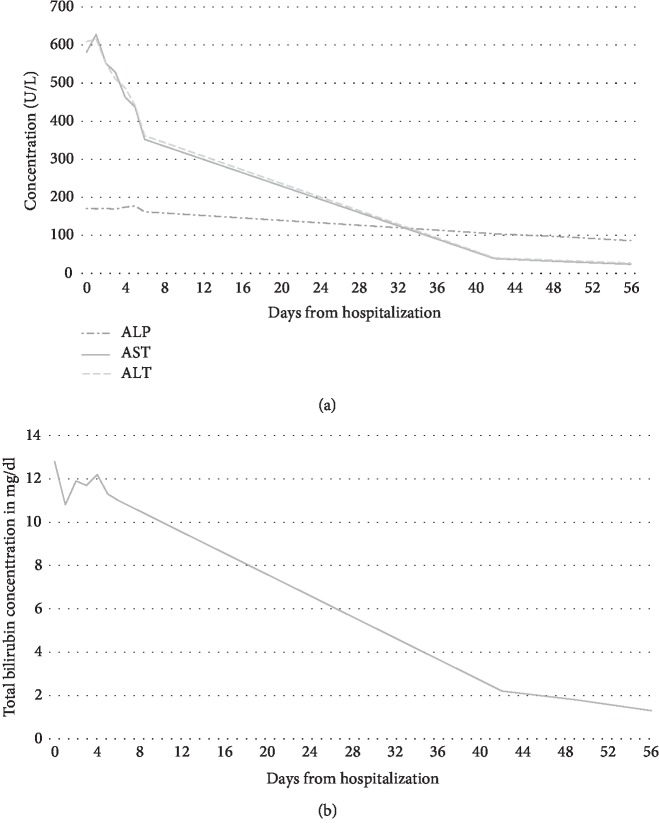
(a) Alkaline phosphatase, aspartate aminotransferase, and alanine aminotransferase levels trend since hospitalization. ALP: Alkaline Phosphatase. AST: Aspartate aminotransferase. ALT: Alanine aminotransferase. (b) Total bilirubin levels trend since hospitalization.

A liver biopsy was planned if no improvement in the patient's liver chemistries was noted. Her liver function tests on day 7 of her admission showed an improvement of  >40% in her ALT and AST, and so a biopsy was deferred. The curcumin supplement was discontinued on admission to the hospital, and the remainder of her medications continued except for simvastatin which wasn't restarted. A follow-up visit 42 days from the initial presentation showed complete resolution of her jaundice, and repeat labs showed normalization of her liver enzymes. The patient was instructed to avoid the curcumin supplement and no re-challenge was performed. No immunosuppressive treatments were initiated at any point during her management. The curcumin supplement the patient was taking was reviewed, and the label reported the following ingredients; 500 mg of curcumin; bovine gelatin; less than 2% of silica; and vegetable magnesium stearate. Follow-up liver enzymes two months and at 18 months from presentation remained normal. A trend of the patient's liver chemistries was summarized in Figures 1(a) and 1(b).

## 3. Discussion

HDS-related DILI is on the rise, with 20% of DILI reactions attributed to HDS in 2010–2012 compared to 7% in 2004–2005 in the U.S. [8]. Herbal product sales continue to rise in the U.S. [4], and while numerous herbs and supplements have been identified as DILI culprits, almost no reports exist on curcumin supplements. This manuscript highlights a case of DILI attributable to a curcumin supplement in a 78-year old female with a latency period of one month.

In the case reported, the patient's R-score was 15 consistent with a hepatocellular pattern of liver injury. DILI is a diagnosis by exclusion, and an extensive evaluation revealed no alternative etiology in our patient. A potential limitation was the lack of hepatitis C virus (HCV) RNA testing, and hepatitis E serologies. Testing for HCV-RNA and hepatitis E is recommended by the European Association for the Study of Liver (EASL) guidelines, particularly when there is no well-established causation between an agent and DILI [9]. Viral hepatitis serologies were negative or suggested previous exposure, the patient had no evidence of shock or hypotension, no evidence of biliary or hepatic pathology on MRCP, no evidence to support hemochromatosis, and autoimmune serologies argued against autoimmune hepatitis as detailed below. Hepatitis E testing was not obtained given the patient had no ill contacts, no recent travel history, and lived in a nonendemic country. The temporal association, and the marked improvement of the liver chemistries with discontinuation of the agent are consistent with the diagnosis of a supplement-induced DILI. An updated Roussel Uclaf Causality Assessment Method (RUCAM) score calculated for the supplement was 6 indicating a probable association (with scores >8 indicating a highly probable category, that is the highest association level on the score) [10].

In numerous reported cases on supplement-induced DILI, the actual supplement is often not identified. In the case reported, the supplement was identified as a supplement produced by a national pharmacy chain, and its label composition was reviewed clarifying the absence of other confounding hepatotoxic ingredients in the supplement. The Food and Drugs Administration (FDA) in the U.S. requests supplement labels to contain information regarding binders, colors, excipients, fillers, flavors, sweeteners, and other active ingredients in supplements. Ideally, a laboratory analysis on the supplement would have helped confirm the absence of inadvertent contaminants.

Lukefahr reported a case of drug induced autoimmune hepatitis attributed to a turmeric containing supplement [11]. In the manuscript discussed, the patient's ANA titer is positive. However, more specific antibodies were unremarkable (ASMA titer < 1 : 40, negative anti-LKM antibodies) and no hypergammaglobulinemia is present. Furthermore, the spontaneous improvement with discontinuation of the supplement, and without any immunosuppressive treatment such as steroids argue against a picture of autoimmune hepatitis.

The pathogenesis of DILI is variable depending on the agent involved and that of herbal and supplement induced liver injury is poorly understood. Mechanisms of hepatotoxicity include; immune mediated; nonimmune; idiosyncratic; and direct hepatotoxicity [12, 13]. The ubiquity of curcumin in various cuisines, the rarity of reported hepatotoxic reactions in the literature, and the absence of convincing evidence for an immune mediated mechanism in the case reported, suggest an idiosyncratic reaction as a possible pathogenetic mechanism for the curcumin-induced DILI. Idiosyncratic reactions are believed to be mediated through the adaptive immune system and involve a complex interaction between predisposing genetic susceptibilities and immune responses [12].

More than two-thirds of patients do not disclose supplement use to their healthcare providers [14]. Numerous factors contribute to this incredibly high percentage including; lack of inquiry by physicians; patients' fears of being judged by their providers; and patients' inaccurate beliefs about the safety of these products [14].

In conclusion, this case illustrates the importance of recognizing curcumin supplements as DILI triggers by healthcare providers. It also reiterates the importance of a detailed evaluation of all over the counter agents and supplements that patients use by primary care providers, hospitalists, and hepatologists to identify potential DILI culprits, and educate patients about the risks involved. Strong relationships of trust between providers and patients, and a nonjudgmental approach to inquiries about complementary medicine use are both essential to allow providers to obtain critical information to prevent inadvertent DILI reactions that can lead to significant morbidity and mortality.
